# Progesterone Is Essential for Protecting against LPS-Induced Pregnancy Loss. LIF as a Potential Mediator of the Anti-inflammatory Effect of Progesterone

**DOI:** 10.1371/journal.pone.0056161

**Published:** 2013-02-07

**Authors:** Julieta Aisemberg, Claudia A. Vercelli, María V. Bariani, Silvia C. Billi, Manuel L. Wolfson, Ana M. Franchi

**Affiliations:** 1 Centro de Estudios Farmacológicos y Botánicos (CONICET-UBA), Buenos Aires, Argentina; 2 Instituto de Investigaciones Biotecnológicas, Universidad de San Martín, Buenos Aires, Argentina; Konkuk University, Republic of Korea

## Abstract

Lipopolysaccharide (LPS) administration to mice on day 7 of gestation led to 100% embryonic resorption after 24 h. In this model, nitric oxide is fundamental for the resorption process. Progesterone may be responsible, at least in part, for a Th2 switch in the feto-maternal interface, inducing active immune tolerance against fetal antigens. Th2 cells promote the development of T cells, producing leukemia inhibitory factor (LIF), which seems to be important due to its immunomodulatory action during early pregnancy. Our aim was to evaluate the involvement of progesterone in the mechanism of LPS-induced embryonic resorption, and whether LIF can mediate hormonal action. Using *in vivo* and *in vitro* models, we provide evidence that circulating progesterone is an important component of the process by which infection causes embryonic resorption in mice. Also, LIF seems to be a mediator of the progesterone effect under inflammatory conditions. We found that serum progesterone fell to very low levels after 24 h of LPS exposure. Moreover, progesterone supplementation prevented embryonic resorption and LPS-induced increase of uterine nitric oxide levels *in vivo*. Results show that LPS diminished the expression of the nuclear progesterone receptor in the uterus after 6 and 12 h of treatment. We investigated the expression of LIF in uterine tissue from pregnant mice and found that progesterone up-regulates LIF mRNA expression *in vitro*. We observed that LIF was able to modulate the levels of nitric oxide induced by LPS *in vitro*, suggesting that it could be a potential mediator of the inflammatory action of progesterone. Our observations support the view that progesterone plays a critical role in a successful pregnancy as an anti-inflammatory agent, and that it could have possible therapeutic applications in the prevention of early reproductive failure associated with inflammatory disorders.

## Introduction

Maternal infection is one of the main causes of spontaneous abortion in humans [1]. In rodents, infection has been associated with an adverse developmental outcome, including embryonic resorption, intrauterine fetal death, intrauterine growth retardation and preterm delivery [2], [3], [4]. In the case of gram-negative bacterial infections, the pathogenic role may result mainly from the presence of the bacterial cell wall component lipopolysaccharide (LPS). Systemic LPS circulation elicits a series of signal transduction events that culminate in the release of numerous biochemical mediators, including cytokines, arachidonic acid metabolites, nitric oxide and toxic O_2_ radicals, among others [5]. Several of these cytokines have been involved in the delicate immune system balance that exists within the feto-maternal interface. Therefore, maternal immune activation induced by LPS may terminate embryo viability. However, the exact mechanism(s) of LPS-induced pregnancy loss remain unclear. We have previously developed a murine model to study the mechanisms of LPS-induced embryonic resorption. In our model, intraperitoneal administration of 1 µg of LPS per gram of body weight on day 7 of gestation produced 100% embryonic resorption at 24 h and expulsion of the resorbed fetus within the next 24 h [3], [4].

Progesterone plays a key role in the reproductive events associated with the establishment and maintenance of pregnancy. The need of progesterone for a successful pregnancy is shown by the fact that blocking hormonal binding sites causes abortion or preterm labor in humans and various animal species [6], [7]. Besides supporting uterine development through its endocrine functions, progesterone acts as an immunosteroid. Progesterone-dependent immunomodulation is one of the mechanisms that enables pregnancy to proceed to term because it protects the “semi-allogeneic” conceptus (due to its paternal antigens, the fetus may be regarded as a semi-allograft in the maternal organism) from immunological rejection. Recent studies suggest autocrine/paracrine factors such as cytokines play a critical role, possibly as effectors of steroid hormones. However, there is still considerable uncertainty about how the action of progesterone is mediated.

LIF is a cytokine with a history of discovery and rediscovery owing to its range of pleiotropic actions on many different cells. Many studies have demonstrated that LIF plays a primary role in embryo implantation, both in humans and mice. It is produced by endometrial epithelial cells, natural killer cells and Th2-like cells. Given that several studies have suggested the importance of some cytokines, particularly LIF, for a successful pregnancy outcome, it is not surprising that changes in the LIF pathway are associated with multiple reproductive pathologies [8], [9].

In addition, LIF, like tumor necrosis factor alpha and interleukin-1, is involved in mediating aspects of the inflammatory response such as stimulation of the acute-phase protein synthesis and the induction of cachexia. It seems that endogenous LIF has anti-inflammatory properties and may even play a regulatory role in the production of several pro-inflammatory cytokines, as has been proposed [10], [11].

The primary aim of the present study was to evaluate the relevance of progesterone in LPS-induced embryonic resorption due to its likely anti-inflammatory effects on maternal uteri. Concerning LIF, our study was restricted to assessing the contribution of this cytokine to hormonal effects.

## Results

### Effect of LPS Treatment on Serum Progesterone Levels

**Figure 1:**
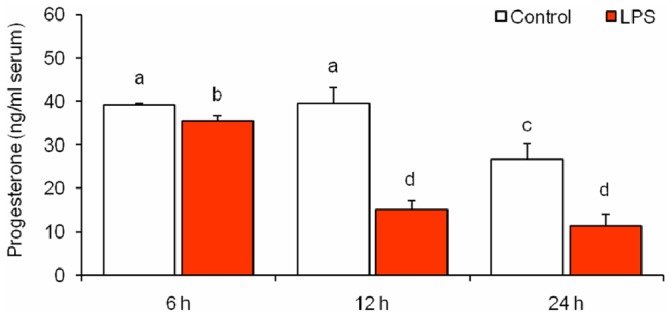
Effect of LPS treatment on serum progesterone levels. Mice were injected on day 7 of gestation and sacrificed at 6, 12 and 24 h after LPS (1 µg/g) or vehicle (PBS) administration. Progesterone was evaluated in serum samples by radioimmunoassay. Progesterone levels are expressed as ng/ml serum. n = 6–9. Values are means ± SEM. Bars with different superscript letters denote significant differences (P<0.05). a≠b≠c≠d.

Considering that there is a close relationship between the amount of circulating progesterone and an ongoing pregnancy, we investigated whether LPS administration could change serum progesterone levels. As mentioned before, the administration of 1 µg/g of LPS on day 7 of gestation produced 100% embryonic resorption at 24 h. Previous results showed that in these mice, implantation sites were necrotized 24 h after LPS administration [4]. Considering this, pregnant mice were treated with either LPS (1 µg/g) or vehicle, and serum progesterone was quantified 6, 12 and 24 h after endotoxin injection. We observed that treatment with LPS was associated with a small reduction in serum progesterone 6 h after the injection. The mean progesterone levels from control animals differed from LPS-treated mice by only 9%; despite this, the difference was statistically significant. The treatment led to a remarkable decline of 60% in serum progesterone after both 12 and 24 h, compared with levels in controls harvested at the same time (Figure 1).

### Effect of Progesterone Supplementation on LPS-induced Embryonic Resorption Rate

Among the pharmacological tools for sustaining pregnancy when there is a threat of abortion, progesterone supplementation represents the most commonly used therapy [12]. As mentioned before, this implies that there exists a direct dependence between circulating progesterone levels and a successful pregnancy. In order to test this, we evaluated the ability of exogenous progesterone to modulate pregnancy outcome in LPS-treated mice, in which serum progesterone declined significantly. Animals on gestational day 7 were pre-treated with progesterone (2 mg/mouse) 2 h before LPS or vehicle injection, and the embryonic resorption rate was evaluated on day 12 of gestation. We found that progesterone supplementation partially prevented embryonic resorption in animals treated with 1 µg/g of LPS (Table 1), even though we administered a pharmacological progesterone dose. Since we used a relatively high dose of LPS to trigger fetal loss, we tested low doses (0.5 µg/g and 0.3 µg/g) that also induce complete embryonic resorption. Animals were pre-treated with progesterone (2 mg/mouse, 2 h before LPS injection) and were then injected with 0.5 µg/g or 0.3 µg/g of LPS. We found that progesterone supplementation partly decreased the proportion of embryonic resorption in mice treated with 0.5 µg/g of LPS. The lowest dose of LPS (0.3 µg/g) produced a resorption rate equal to control values. The results are shown in table 1.

**Table 1:** Embryonic resorption rate in mice pre-treated with progesterone.

Experimental groups	ER% (mean ± SEM)
Control (n = 12)	6,6±2,1 **[a]**
LPS (**1 µg/g**) (n = 8)	100,0±0,0 **[b]**
LPS (**0,5 µg/g**) (n = 8)	100,0±0,0 **[b]**
LPS (**0,3 µg/g**) (n = 6)	87,5±12,5 **[b]**
Progesterone (2 mg/mouse)+LPS (**1 µg/g**) (n = 6)	59,8±11,6 **[c]**
Progesterone (2 mg/mouse)+LPS (**0,5 µg/g**) (n = 6)	51,5±21,7 **[c]**
Progesterone (2 mg/mouse)+LPS (**0,3 µg/g**) (n = 10)	24,2±15,5 **[a]**

Different letters (in brackets) denote significant differences (P<0.05). a≠b≠c.

Groups of mice were injected with LPS (1, 0.5 and 0.3 µg/g) and LPS+progesterone (2 mg/mouse, 2 h before LPS injection) on day 7 of gestation. Embryo resorption rates were determined on day 12 of gestation and are expressed as percentages (number of dead embryos/(number of dead embryos+number of healthy embryos) X 100). Different letters (in brackets) denote significant differences (P<0.05). a≠b≠c.ER: embryonic resorption.

After five days of LPS administration on day 7 of gestation, the uterus from treated mice showed no sign of having been pregnant because LPS treatment led to 100% embryonic resorption. In the uterus from mice treated with LPS and progesterone we observed embryos in various stages of resorption. Some of them were healthy, other underdeveloped and with a marked difference in size. Some hemorrhagic areas were observed, in which the shape of a fetus could not be recognized.

### Effect of Progesterone Supplementation on Uterine Nitric Oxide Levels

**Figure 2:**
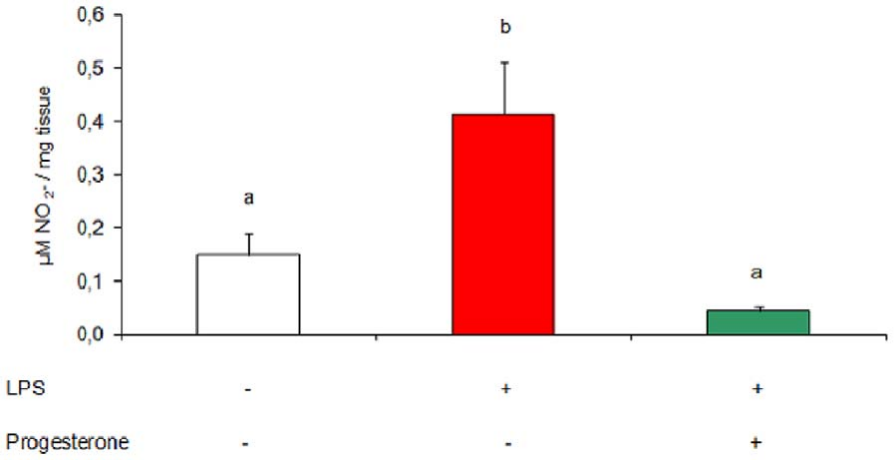
Effect of LPS and progesterone treatment on uterine nitric oxide levels. Mice were injected on day 7 of gestation with LPS (1 µg/g) or LPS and progesterone (2 mg/mouse, 2 h before LPS injection). They were sacrificed 6 h after LPS treatment and uterine tissue was cultured for 24 h. Nitric oxide was measured as nitrate (NO_3_^−^) plus nitrite (NO_2_^−^) accumulation in culture supernatants by Griess technique. n = 5. Values are means ± SEM. Results are expressed as µM NO_2_^−^/mg tissue. Bars with different superscript letters denote significant differences (P<0.05). a≠b.

As we mentioned above, progesterone acts as an immunomodulator and exerts anti-inflammatory effects throughout pregnancy. It has been proposed that optimal levels of nitric oxide are needed for a successful pregnancy. However, excessively high amounts are shown to impair embryo implantation, causing embryonic resorption processes. Nitric oxide synthesis occurs in vast amounts during infection-induced pregnancy loss [4]. To evaluate the protective action of progesterone, we assessed nitric oxide levels in uteri from mice treated *in vivo* with progesterone. Animals were injected with vehicle, LPS (1 µg/g) or LPS and progesterone (2 mg/mouse, 2 h before LPS injection) on day 7 of gestation and were sacrificed 6 h after endotoxin administration. Nitric oxide produced by uterine tissue was measured as the accumulation of nitrate (NO_3_^−^) and nitrite (NO_2_^−^) in culture supernatants by Griess technique. We observed that LPS induced the augmentation of nitric oxide levels in the uterus, as we had already found in a previous work [4]. Also, we observed that progesterone supplementation reduced nitric oxide levels to control values (Figure 2). This result suggests that progesterone may have an anti-inflammatory effect, since it was capable of down-regulating nitric oxide levels.

### Effect of LPS and Progesterone Treatment on PR Expression in Uterus

**Figure 3:**
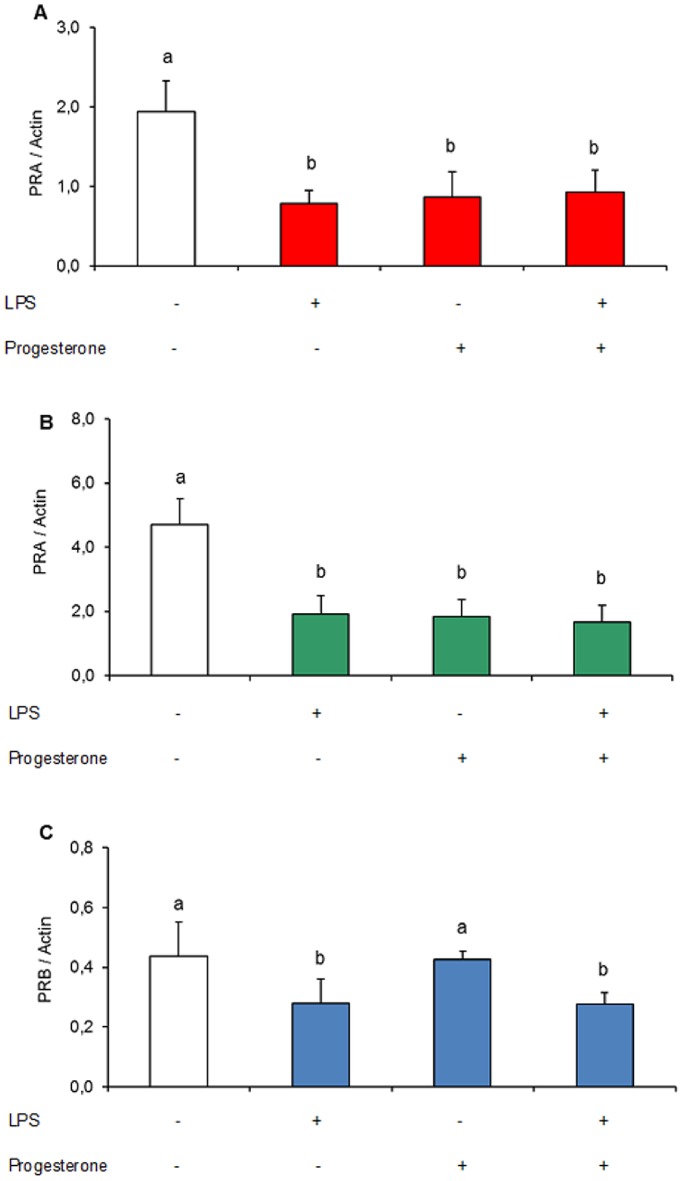
Effect of LPS treatment on progesterone receptor (PR) protein levels in uterus. Mice were injected on day 7 of gestation with vehicle (PBS), LPS (1 µg/g), progesterone (2 mg/mouse) or LPS+progesterone and sacrificed at 6 and 12 after treatment. PRA and PRB protein expression were evaluated in uterine samples by western blot. (**A**) PRA 6 h, (**B**) PRA 12 h and (**C**) PRB 6 h. PR levels are expressed as relative optical density (PR/Actin). n = 6–5. Values are means ± SEM. Bars with different superscript letters denote significant differences (P<0.05). a≠b.

The ratio of PR isoforms changes during pregnancy and reflects different progesterone effects on reproductive tissues. Our aim was evaluate the early pregnancy expression in uterus of the two main types of nuclear PR in mice and whether their levels change as inflammation advances or by progesterone supplementation. Animals were treated with LPS, progesterone or both on day 7 of gestation and sacrificed 6 and 12 h after treatment. PRA and PRB were evaluated by western blot. First, we identified the expression of PRA and PRB in pregnant mice uterus by day 7. PRB is the principal mediator of progesterone action whereas PRA represses the transcriptional activity of PRB. We found that levels of PRB diminished after 6 h of LPS treatment. In the uterus of mice treated during 12 h with LPS or progesterone or both, the expression of PRB decreased markedly (data not shown). In most of the western blots that were made, PRB was almost undetectable and in a few, the band which corresponds to PRB was very dim. In addition, we observed that PRA expression diminished significantly after 6 and 12 h of treatment. This occurred regardless of the treatment concerned (Figure 3).

### Distribution of PR and LIF in the Uterus of Pregnant Mice

**Figure 4:**
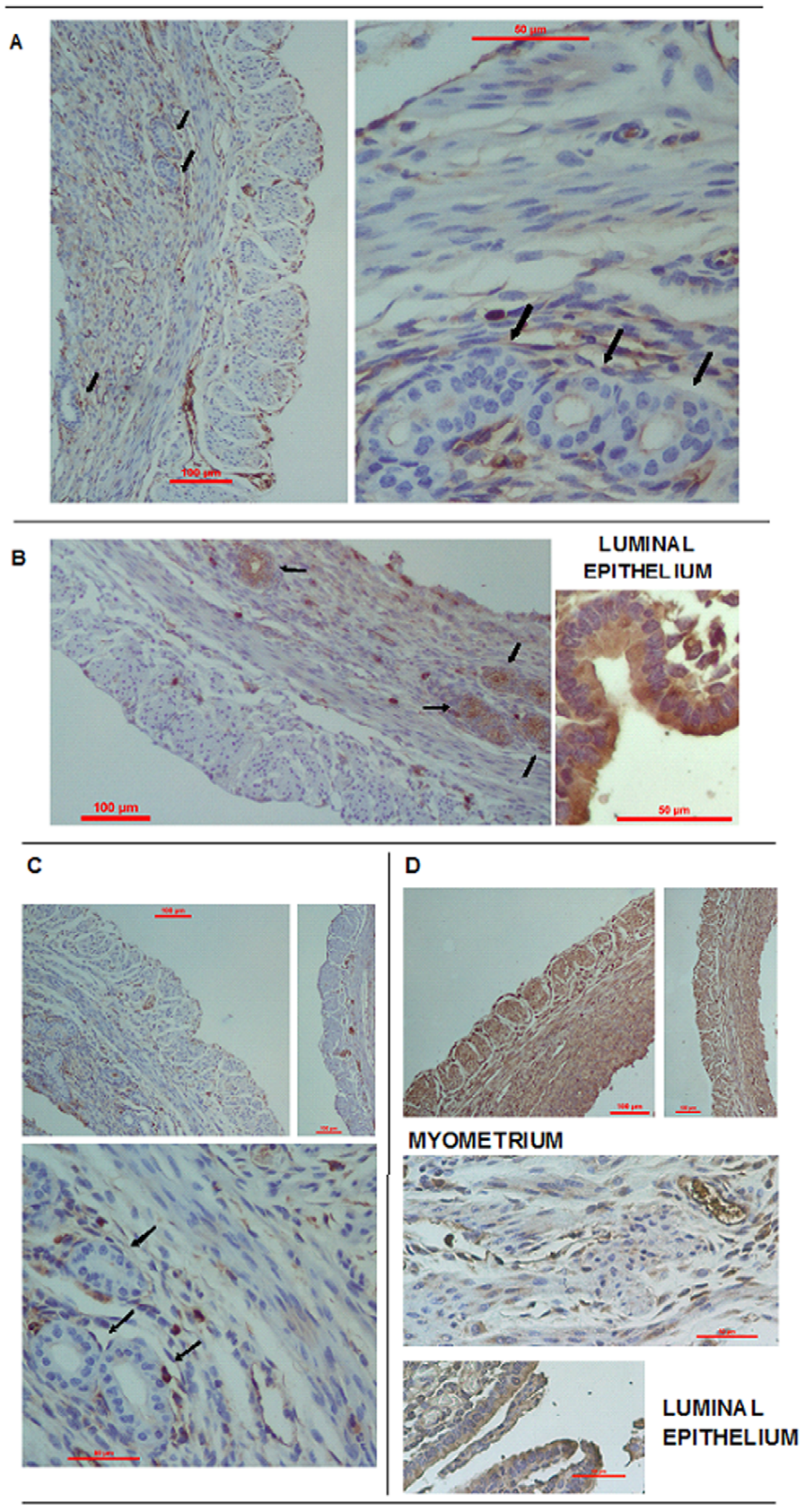
Immunolocalization of PR (A) and LIF (B) in uteri from day 7 of pregnancy in mice. Tissue sections were processed by the immunoperoxidase technique using specific antibodies. (**A**) Negative controls for LIF, (**B**) Immunostaining for LIF, (**C**) Negative controls for PR and (**D**) Immunostaining for PR. Arrows indicate glands. The scale bar indicates 10 µm. Magnifications: x40 and x100.

PR and LIF cellular localization were measured by immunohistochemistry in the uterus of control animals on day 7 of pregnancy. PR immunoreactivity was detected in luminal and glandular epithelium, as well as in myometrial cells and endometrial stroma (Figure 4D). Furthermore, the protein was distributed abundantly throughout the mesometrial and antimesometrial decidua (data not shown). The results revealed that both luminal and glandular epithelial cells abundantly expressed LIF (Figure 4B). Moreover, the stromal component of the endometrium showed LIF staining. The adjacent decidua exhibited a strong expression of LIF (data not shown). The myometrium was devoid of immunostaining for the LIF protein. Particularly, a diffuse and granular cytoplasmic staining pattern was observed in cells. The results observed here regarding the location of PR and LIF in the uterus are consistent with those published until now [13], [14], [15], [16], [17], [18], [19], [20].

### Effects of LPS and Progesterone on Uterine LIF mRNA Expression *in vitro*

**Figure 5:**
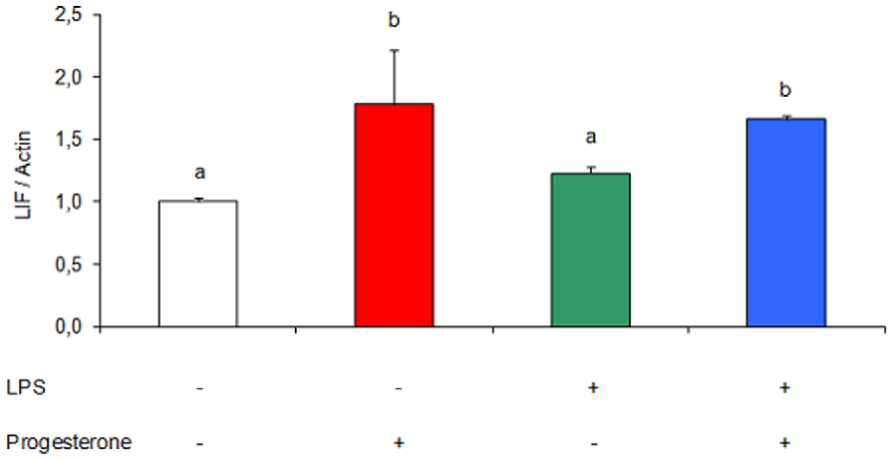
Effect of progesterone on uterine LIF expression. Mice were sacrificed on day 7 of gestation and uterine tissue was cultured for 2 h with and without LPS (1 µg/ml) and progesterone (50 ng/ml). LIF mRNA levels was determined by RT-PCR. n = 5. Values are means ± SEM. Results are expressed as relative optical density (LIF/Actin). Bars with different superscript letters denote significant differences (P<0.05). a≠b.

It is well known that LIF could be regulated by ovarian hormones. With the aim of evaluating the progesterone regulation of LIF, we designed an *in vitro* experiment. Mice were sacrificed on day 7 of gestation and after uterus isolation the tissues were cultured for 2 h in wells that contained LPS (1 µg/ml, [21]) and progesterone (50 ng/ml), alone or in combination. After that, we determined LIF mRNA expression in uterine explants. We observed that progesterone incubation stimulated LIF mRNA expression at the time analyzed (Figure 5).

### Effects of Exogenous LIF on Uterine Nitric Oxide Levels *in vitro*

**Figure 6:**
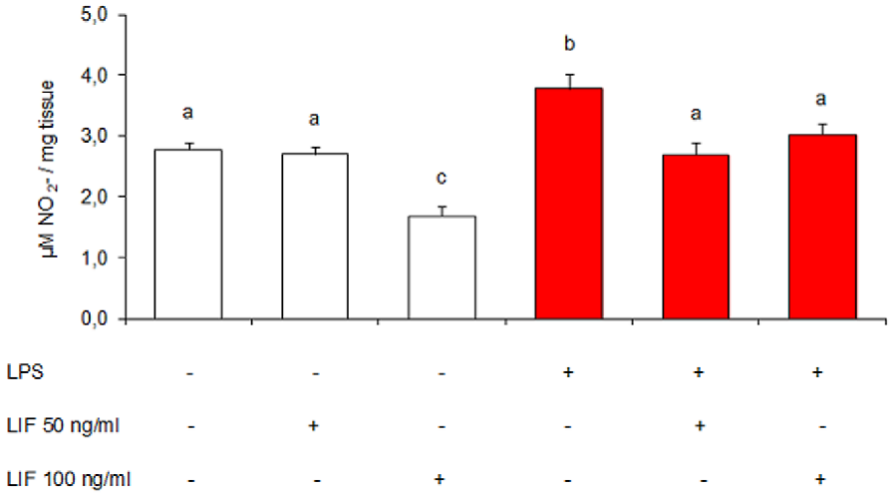
Effect of LPS and exogenous LIF on uterine nitric oxide levels. Mice were sacrificed on day 7 of gestation and uterine tissue was cultured for 24 h. Assays were performed using LPS (1 µg/ml) and murine recombinant LIF (50 and 100 ng/ml). Nitric oxide was measured as the accumulation of nitrate (NO_3_^−^) and nitrite (NO_2_^−^) in culture supernatants by Griess technique. n = 6. Values are means ± SEM. Results were expressed as µM NO_2_^−^/mg tissue. Bars with different superscript letters denote significant differences (P<0.05). a≠b≠c.

LIF can act in both pro-inflammatory and anti-inflammatory ways, depending on a number of variables. Intending to elucidate the role of progesterone and LIF in our model of pregnancy loss, uteri from non-treated mice (on day 7 of gestation) were cultured with/without LPS (1 µg/ml) and murine recombinant LIF (50 and 100 ng/ml). Nitrite accumulation was assessed in culture supernatants. As we observed *in vivo* (Figure 2), LPS stimulated nitric oxide levels *in vitro*. We found a significant decrease in nitrite levels when LPS and LIF were added simultaneously to cultures (Figure 6). This result suggests the ability of LIF to mimic the progesterone-associated anti-inflammatory action.

### Effect of Endogenous LIF on Uterine Nitric Oxide Levels *in vitro*

**Figure 7:**
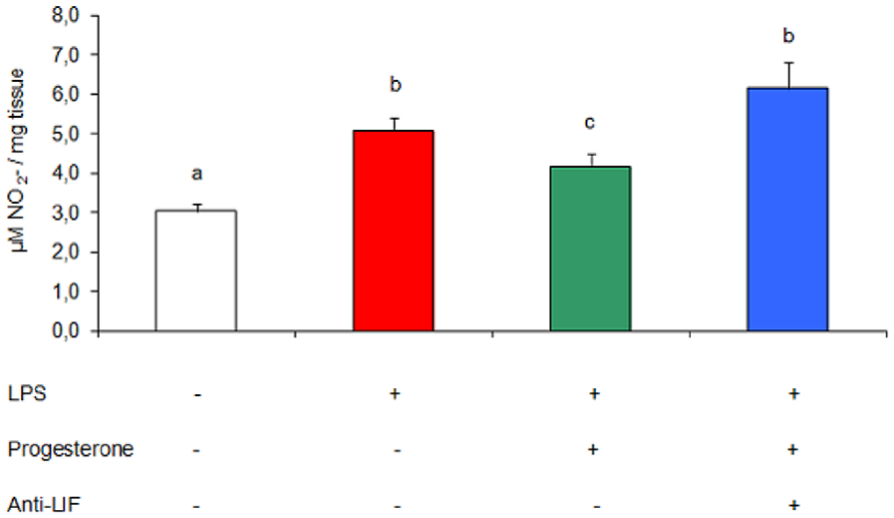
Effect of endogenous LIF and progesterone on uterine nitric oxide levels. Mice were sacrificed on day 7 of gestation and uterine tissue was cultured for 24 h. Assays were performed using progesterone (50 ng/ml), LPS (1 µg/ml) and an antibody raised against murine LIF (anti-LIF: 5 µg/ml). Nitric oxide was measured as the accumulation of nitrate (NO_3_^−^) and nitrite (NO_2_^−^) in culture supernatants by Griess technique. n = 5. Values are means ± SEM. Results were expressed as µM NO_2_^−^/mg tissue. Bars with different superscript letters denote significant differences (P<0.05). a≠b≠c.

With the purpose of evaluating whether the progesterone effect could be mediated by endogenous LIF, uteri from non-treated mice (on day 7 of gestation) were incubated for 24 h with LPS (1 µg/ml), alone or in combination with progesterone (50 ng/ml) and an antibody against murine LIF (5 µg/ml). Then we determined nitrite accumulation in culture supernatants. Co-incubation with progesterone partially decreased nitric oxide production induced by the addition of LPS. The co-incubation with the antibody that neutralizes endogenous LIF abolished the effect of progesterone on nitrite accumulation (Figure 7). This result suggests that uterine LIF probably participates in the protective effect of progesterone, controlling the harmful levels of nitric oxide stimulated by LPS.

### Effect of RU-486 on Uterine Nitric Oxide Levels *in vitro*

**Figure 8:**
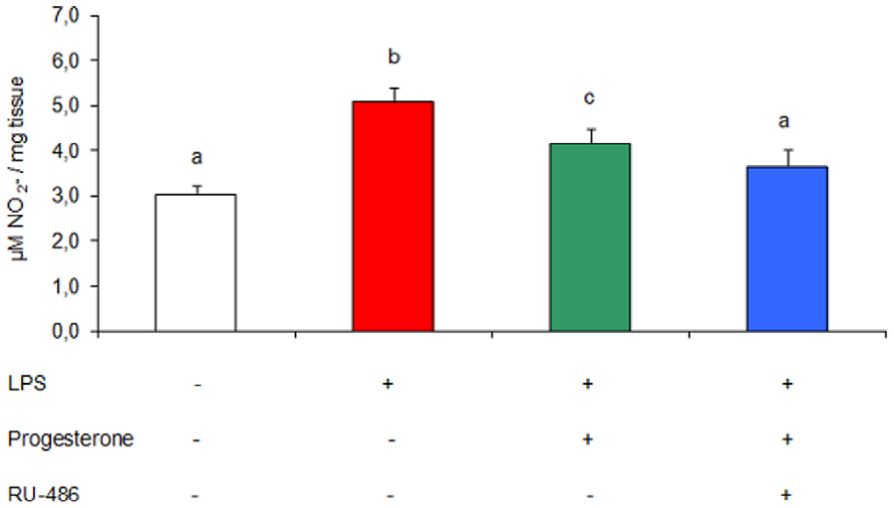
Effect of RU-486 on uterine nitric oxide levels. Mice were sacrificed on day 7 of gestation and uterine tissue was cultured for 24 h. Assays were performed using progesterone (50 ng/ml), LPS (1 µg/ml) and RU-486 (0.1 µM). Nitric oxide was measured as the accumulation of nitrate (NO_3_^−^) and nitrite (NO_2_^−^) in culture supernatants by Griess technique. n = 11. Values are means ± SEM. Results were expressed as µM NO_2_^−^/mg tissue. Bars with different superscript letters denote significant differences (P<0.05). a≠b≠c.

Within the steroid receptor superfamily, progesterone receptor (PR) and glucocorticoid receptor (GR) share regions of high homology, particularly within the DNA binding domain. Progesterone is known to signal via PR and/or GR. In order to explore the down-regulating mechanisms of uterine levels of nitric oxide by progesterone, we tested the effects of RU-486, a drug considered to be a potent progesterone-receptor antagonist, knowing that RU-486 is also a GR antagonist [22]. Uteri from non-treated mice (on day 7 of gestation) were cultured for 24 h and nitrite accumulation was assessed in culture supernatants. The co-incubation with LPS (1 µg/ml), progesterone (50 ng/ml) and RU-486 (0.1 µM) restored nitric oxide production to control values (Figure 8). This result suggests that progesterone could have an anti-inflammatory effect through both PR- and non-PR-mediated mechanisms, but more studies are necessary for elucidating this specific progesterone action pathway.

## Discussion

Progesterone, regarded as the pregnancy hormone, coordinates a series of complex events that ultimately lead to the synchronized development of the embryo and the differentiation of uterine cells for implantation. Decreased progesterone levels, such as what can be experimentally induced by ovariectomy or RU-486 treatment in rodents, leads to abortion [23], [6].

Our results suggest that LPS induces complete embryonic resorption through a mechanism involving deficiencies in serum progesterone levels. We found that serum progesterone level was significantly lower at 6, 12 and 24 h after LPS treatment compared with mice receiving vehicle. An early decrease (6 h), with a subsequent pronounced decline in the levels of this steroid, led to complete pregnancy loss within 24 h. This allowed us to conclude that the administration of 1 µg/g of LPS to mice during early pregnancy is associated with a decrease in serum levels of progesterone that leads to pregnancy loss. This is consistent with previous findings showing that serum progesterone decreases to very low levels after intrauterine bacterial exposure [24] or intraperitoneal administration of LPS [25].

Unlike Agrawal *et al.* [26] we did not observe differences between ovaries from control and treated mice 24 h after LPS injection (data not shown). Differences in the pattern of follicular development or signs of luteal regression were not detected. It should be taken into account that luteal regression is a two-phase process, the first of which, or functional, is associated with the loss of the capacity of the luteal cells to produce progesterone. The second phase, or structural, occurs after the initial decline in progesterone output and involves the morphological involution of the luteal structure. Although in some species both events occur simultaneously [27], in others the corpus luteum first loses its ability to secrete large amounts of progesterone and then degenerates [28]. Although the ability of the corpus luteum to secrete progesterone drops markedly at the end of pregnancy, no sign of severe apoptosis can be found at this time. It is only after parturition that a large increase in the number of luteal cells undergoing apoptosis and a significant decrease in the mass of the gland can be observed [29], [30]. Preliminary results suggest that this is what would happen in mice. We found that at some times before expulsion of the resorbed embryos, when progesterone levels were significantly reduced, the tissue and the cellular structure of the ovary were intact. We believe that the dissociation between the decrease in luteal progesterone production and luteal cell death is what happened in our model. Because of this, although there was an increase in the levels of prostaglandins [3], and circulating progesterone decreased significantly, these effects did not cause structural changes detectable when the analysis was made.

Since LPS-binding sites have been detected in human granulosa/luteal cells [31], we cannot rule out a direct action of LPS on steroid metabolism. Furthermore, Adashi *et al.* [32] have suggested the ability of TNF-α to diminish the gonadotropin-supported accumulation of progesterone by murine granulose/luteal cells. We have previously observed that serum level of TNF-α increases rapidly after LPS treatment (data not shown). Perhaps both effects may contribute to the regulation of circulating progesterone levels in mice treated with LPS.

In our model of early pregnancy loss, progesterone supplementation directly affected the resorption process. A single dose of progesterone prior to the administration of 1 or 0.5 µg/g of LPS partially decreased the rate of embryonic resorption. At the lowest dose of LPS, the embryonic resorption rate returned to control values in animals pre-treated with progesterone. In these latter mice we also tested the full-term delivery. Although treatment with progesterone allows the pregnancy proceed to term, in most cases the pups were born alive but died after birth. It has been reported that an increase in the pro-inflammatory cytokine detected in the fetal brain following injection of LPS is attributable to the production of cytokines by the fetal brain itself [33]. Although progesterone appears to have a protective role by blocking the activation of pathways associated with embryonic resorption, it may not be sufficient to protect the fetus from injury produced by LPS. Our results suggest that progesterone may be a suitable therapeutic tool and that the beneficial effects that may occur would be proportional, in part, to the magnitude of the inflammatory stimulus that causes pregnancy loss.

Many functions of progesterone are mediated through the binding to its specific nuclear receptor (PR) which acts as a ligand-transcription factor. PR exists as two major types: the full-length PRB and the N-terminal truncated PRA, which are transcribed from two different promoters in the same gene. PRA is generally acknowledged to act as a transcriptional inhibitor of PR-B [34]. The relative expression of the two isoforms is conserved in rodents and humans, but varies dramatically between different tissues, cell types, physiological and disease states [35], [36]. We observed a decreased PRA expression in the uterus after 6 and 12 h of any treatment when compared to control values. This is consistent with some results obtained by Agrawal *et al.* [37], who found that PRA (mRNA) expression was very low in uteri recovered from LPS-treated animals on day 4.5 of gestation. They also observed that LPS elevates PRA expression on day 1.5 of pregnancy but attributed this to the increase of estradiol levels in serum. It is well known that estradiol is an activator of PR expression [38], [39], [40]. In the work of Agrawal, as in our own, it was found that LPS decreased circulating progesterone. Marriot and col. [41], reported that infusion of LPS into the female rat brain induces a neuro-inflammatory response which increases the expression of PRA and PRB proteins in the uterus. But this was found in a model of LPS-induced inflammation in ovariectomized rats with an estrogen replacement regimen. In this case, the high levels of estrogens positively modulate PR expession. In contrast to this, our results show that LPS reduced PRB levels in the uterus after 6 h of treatment and were almost undetectable after 12 h. We believe that the inflammatory status induced in these mice by LPS administration provokes a down-regulation of PRB expression, which is the primary PR that mediates the progesterone effects. Furthermore, it is known that progesterone can modulate the expression of its own receptor [42], [43]. Apparently, this happened in our mice, in which progesterone treatment diminished PRA expression in pregnant mice uteri. Particularly, this occurred in a uterus which was subject to a gestational status. Finally, we believe that after 12 h of treatment, the resorption process induces alterations in regulatory signals.

A growing body of evidence suggests that progesterone plays a significant role in the establishment of an adequate immune response during the early stages of pregnancy. A direct relationship between the defective production of LIF and pregnancy loss is associated with recurrent implantation failure [44], [45]. It has been reported that T cells from recurrent aborter women secrete less LIF than those from normal women [46]. Infertile women also have lower levels of expression of endometrial LIF [45].

Murine LIF appears to be regulated *in vivo* by estradiol [15], whereas studies carried out in rabbits, monkeys and humans indicated the role played by progesterone in regulating endometrial LIF [15], [47], [48]. Studies in mice show how the hormonal regimen with estradiol up-regulates the expression of LIF in animals undergoing delayed implantation or in ovariectomized mice under hormonal priming. These results were of great importance, since it is indisputable that LIF is essential for implantation in mice. Here, we evaluated the expression of LIF protein *in vivo*. LIF levels appear to be higher in the uterus from progesterone treated animals, but the difference, compared to control values, was not statistically significant (data not shown). Studies have emphasized until now that although steroid hormones play an important role in the regulation of LIF in the endometrium, the normal action of several other cytokines and growth factors, as well as the interactions between them, are also critical to the *in vivo* modulation of LIF [49]. Therefore, it is believed that the effects of steroid hormones on endometrial LIF appear to be complex and depend on several factors.

Here we have shown that progesterone stimulates uterine LIF expression *in vitro*. Several *in vitro* studies were conducted in order to assess the hormonal regulation of LIF. However, most of them were performed with human endometrium or human endometrial cells [49]. Therefore, our result seems to be the first evidence of the modulation of LIF by progesterone in mice.

Evidence of the modulation exerted by LPS on LIF refers to studies performed *in vivo*, in which the animals were directly injected with E. coli or LPS. Brown *et al.* [50] showed evidence of the induction of LIF in mouse lung explants. However, the tissue came from mice treated with LPS *in vivo*. There are no *in vitro* assays demonstrating a modulation of LIF by LPS. In this study, we did it with uteri from pregnant mice and found no changes in mRNA expression of LIF in the presence of LPS.

One of the aims of this study was to evaluate a possible involvement of LIF in mediating the anti-inflammatory effect of progesterone. We tested in an *in vitro* assay whether the presence of LIF produces the same effect as the presence of progesterone on the uterus of a pregnant mouse. We found that the addition of either LIF or progesterone has the same anti-inflammatory effect, reducing uterine nitrite levels. However, it seemed more important to assess whether endogenous LIF (from the maternal uterus) could modulate nitric oxide levels. First, we found that, as observed *in vivo*, progesterone down-modulated the nitric oxide levels *in vitro*. Blocking the action of endogenous LIF in the presence of progesterone, it increases nitric oxide levels, matching them to the levels observed upon incubation with LPS only. We propose that uterine LIF could act as an intermediary in the action of progesterone and could be an important mediator for controlling infections during early pregnancy.

We believe that the anti-inflammatory effect of progesterone may be one of the reasons by which this hormone is decisive for the establishment and maintenance of gestation. In addition, this effect seems to be one of the causes why this steroid can reduce the risk of pregnancy loss. These conclusions are reinforced by the results obtained from an *in vivo* assay which evaluated the levels of nitric oxide in the uterine tissue from mice treated with progesterone and LPS. One of the contributions of this study is to demonstrate that progesterone plays an anti-inflammatory role by reducing nitric oxide levels induced by LPS both *in vivo* and *in vitro*.

We have previously shown that pregnant uterus from mice treated with LPS exhibits biochemical and histological characteristics of inflammation (there is an increase in the levels of nitric oxide and prostaglandins, and also infiltration of immune cells at implantation sites as a consequence of LPS injection [3], [4]) accompanying the progress of an active pregnancy loss. It is known, and has been confirmed in this study, that PRs are expressed in the epithelial, stromal and myometrial compartments of the uterus. We assume that the PRs that are located in these tissues are jointly involved in the process of pregnancy loss. A function of progesterone that is crucial for pregnancy and is related with myometrial cells is the maintenance of uterine quiescence. PRs are especially important because inhibition of its activity increases uterine excitability and contractility [51], [52], [53]. Progesterone regulates the expression of various genes associated with myometrial contractility as receptors for stimulatory uterotonins, prostaglandin metabolic enzymes and gap-junction proteins. In rodents, progesterone decreases myometrial responsiveness to oxytocin and PGF_2α_ by inhibiting their receptor expression [54], [55]. We have previously demonstrated that LPS administration activated nitric oxide and prostaglandins pathways, which leads to an increase of these mediators in the maternal-fetal interface [3], [4]. This allows us to speculate about the fundamental involvement of myometrial PR in the mechanism of resorption, particularly in the loss of uterine relaxation. The latter is evident by the fact that dead embryos were expelled within 48 h after LPS administration to the mother.

The capacity for progesterone to affect gene expression was dependent on the PR-A to PR-B ratio, especially for the expression of pro-inflammatory genes. Progesterone decreased pro-inflammatory gene expression when the PR ratio favored PR-B and increased pro-inflammatory gene expression when favored PR-A. Progesterone via PR-B increased expression of inhibitor-κBα (a repressor of the nuclear factor-κB transcription factor) and inhibited basal and stimulated pro-inflammatory gene expression [56], [57]. When myometrial cells are PR-B dominant, progesterone promotes myometrial quiescence. The anti-inflammatory effect mediated by PRB also occurs at stromal and epithelial cells. Interestingly, we found that as the infection progresses PRB expression decreases to undetectable levels. Even though PRA also decreases, never disappears at the time evaluated. We suppose that the expression of PRA, even though diminished, promotes pro-inflammatory signals in the tissues that surround and interact with the embryo.

Several cytokines, chemokines and growth factors are produced by the endometrial glands and secreted into the uterine cavity where they act both on the blastocyst and on the endometrial surface, changing adhesive capacity, modifying blastocyst development and outgrowth and providing chemoattraction, in addition to functions in immune regulation [58], [59], [60]. Therefore, progesterone receptors located in the glandular epithelium also have a fundamental contribution to the establishment and maintenance of the pro-inflammatory intrauterine environment.

We previously found that LPS induced a marked leukocyte infiltration at implantation sites. The increase in the number of activated lymphocytes and macrophages in response to LPS in the reproductive tissues adversely affects the embryonic development. Therefore, LPS-induced inflammation leads to changes in the maternal immune system action (cytokine environment of Th2 dominant to a Th1 profile) characterized by the synthesis and secretion of pro-inflammatory cytokines. Regarding this, is important to note that LIF is produced by endometrial epithelial cells and also by natural killer cells and Th2-like cells. This potential effect of dysregulation of such mechanisms on the maternal immune response could result in pregnancy loss.

In this study we observed LIF protein expression in epithelial and stromal cells of the uterus, which is consistent with those of available background information [13], [14], [17], [61]. In rodents, several factors including LIF, are produce exclusively by endometrial glands and are critical for conceptus survival, development and implantation. The primary role of LIF in implantation was first demonstrated by studies in which transgenic mice homozygous-deficient for LIF could produce normal embryos, but implantation failed to occur [62]. It should be noted that in our model we injected LPS on day 7 of gestation, nearly three days after implantation has occurred. This suggests that LIF in treated mice could act mainly as a post-implantatory immunomodulator involved in regulating of the inflammatory response and in the mechanisms of tolerance to the fetus.

Our data suggest the possibility that the early stages of pregnancy may be particularly sensitive to progesterone deficiency. If the decrease of systemic progesterone is one of the main mechanisms by which inflammation induces pregnancy loss, our results reinforce the benefit of using progesterone to reduce the risk of miscarriage. Based on these findings, we suggest that the functional network between hormones, cytokines and hormonal mediators at the feto-maternal interface has a fundamental role in the development of a successful pregnancy. A defect in the integrity of this network probably leads to pregnancy loss.

## Materials and Methods

### Animals and Treatments

Virgin female mice of the *BALB/c* strain (8- to 12-week-old; 25–30 g of weight) were paired with adult *BALB/c* males; the day of appearance of the post-coitum vaginal plug was taken as day 0 of gestation. Animals received food and water *ad libitum* and were housed under controlled conditions of light (12 h light/12 h dark) and temperature (23–25°C). Mice were euthanized by CO_2_ inhalation.

On day 7 of gestation the animals were divided randomly into three groups for the *in vivo* experiments and treated respectively with LPS (1 µg/g, i.p) or LPS and progesterone (2 mg/mouse, s.c., 2 h before LPS administration). Progesterone and LPS were dissolved in corn oil or phosphate buffered saline (PBS), respectively. Mice were injected (0.1 ml) at 9∶00–10∶00 on day 7 of gestation and were sacrificed at different times after injection. In mice, the endometrial estroma undergoes dramatic cytologic changes to form a decidua in response to the implanted embryo. After attachment, the embryo becomes embedded in an enlarging mass of decidual tissue, and this tissue is separated from uterine adventitia by an inner circular and an outer longitudinal smooth muscle layer (myometrium). After isolation of each implantation site the uterus was cut open along the longitudinal axis. Decidual tissues were separated from the myometrium by gently scrapping off the decidua. Serum and uteri from mice were collected and stored at −80°C until used. Ovaries and the entire implantation sites were extracted and immediately fixed in Formol 4% for hematoxilin-eosin histological staining and immunohistochemistry, respectively.

For the *in vitro* experiments, non-treated mice were sacrificed at 9∶00–10∶00 on day 7 of gestation and their uteri were used for culture assays. *In vitro* assays were performed using LPS: 1 µg/ml [21]); progesterone: 50 ng/ml (0.16 µM; see materials and methods section); murine recombinant LIF: 50 and 100 ng/ml [63], [17]); anti-LIF antibody: 5 µg/ml [64]; RU-486∶0.1 µM [65].

### Ethics Statement

The experimental procedures reported here were approved by the Animal Care Committee of the Center for Pharmacological and Botanical Studies of the National Research Council (CEFYBO - CONICET) and by The Institutional Committed for the Care and Use of Laboratory animals from the School of Medicine (University of Buenos Aires), and were carried out in accordance with the Guide for Care and Use of Laboratory Animals (NIH).

### Determination of Embryonic Resorption Rate

With the aim of assessing the rate of embryonic resorption, mice were treated on day 7 and sacrificed on day 12 of gestation. The uteri were excised and examined macroscopically to count the number of healthy and resorbed embryos. The resorbed embryos were identified by their small size, extensive hemorrhage and necrosis. We consider that an embryo that contains any of these features is an embryo reabsorbed. Resorption rates were calculated as: number of dead embryos/(number of dead embryos+number of healthy embryos)×100.

### Western Blot Analysis

Uteri from pregnant mice treated with vehicle, LPS, progesterone or LPS+progesterone were homogenized (Ultra Turrax, T25 basic, IKA Labortechnik) in lysis buffer (10 mM Hepes, 5 mM MgCl_2_, 142.5 mM KCl, 0.1% SDS, 1% Nonidet-40, 5 mM EDTA, 0.5% Sodium Deoxycholate in phosphate buffered saline) with a freshly added protease inhibitor cocktail (10 µg ml^−1^ leupeptin, 2 µg ml^−1^ aprotinin, 100 µg ml^−1^ soybean-trypsin inhibitor, 1 mmol l^−1^ EDTA, 1 mg ml^−1^ benzamidine, 10 µg ml^−1^ DTT and 1 mg ml−1 caproid acid). Tissues were sonicated (Ultrasonic Cell Disrupter, Microson, Heat systems Inc.) for 30 s, centrifuged at 12000 g for 20 min and protein concentration determined by the Bradford assay [66]. Proteins (80 µg per lane) were separated by electrophoresis in 6–12% or 7.5–15% SDS-PAGE gel (for PR and LIF respectively) and transferred to a nitrocellulose membrane. Membranes were blocked using 5% w/v dried non-fat milk and then incubated with the primary antibodies: PR (1∶150), LIF (1∶150) and β-actin (1∶4000). They were washed with PBS-T (10 mM Tris, 100 mM NaCl and 0.1% Tween 20, pH 7.5) followed by 1 h incubation with horse radish peroxidase-conjugated anti-rabbit secondary antibody (1∶3000) and developed using the enhanced chemiluminescence system. The images for immunoreactive bands were acquired using the ImageQuant blot documentation instrument and analyzed using the Image J software package. Results were expressed as relative optical density (PRA, PRB, or LIF/β-actin).

### Immunohistochemical Analysis

Implantantion sites from pregnant mice on day 7 of gestation were removed and fixed in formol 4% in phosphate-buffered saline. The tissue sections were dehydrated and embedded in paraffin. Sections of 5 µm were made with a microtome and mounted on 2% silane-coated slides. The sections were stained with hematoxylin–eosin, and observed by light microscopy (Nikon Eclipse 200, NY, USA) to evaluate tissue morphology and cell types. For immunohistochemistry studies, the samples were blocked of endogenous peroxidase and then pre-incubated with blocking buffer (0.1% bovine serum albumin in PBS) in a humidity chamber at room temperature for 1 h. Then, the slices were incubated at 4°C overnight in a humidity chamber with a rabbit antibody directed against PR or LIF (monoclonal 1∶150 and polyclonal 1∶250, respectively). Negative controls were incubated with a purified rabbit antiserum at the same concentration that primary antibodies. The antigen was revealed by diaminobenzidine. Finally, the sections were dehydrated, counterstained and mounted for observation.

### Culture of Uterine Explants

Uterine explants were weighed and cultured in wells containing DMEM (without phenol red) supplemented with 10% fetal calf serum and antibiotics (1%): 20 IU/ml penicillin G, 20 µg/ml streptomycin and 50 ng/ml amphotericin B. Where indicated, tissues were maintained for 2 and 24 h in 5% CO_2_ in air at 37°C and then supernatants and explants were immediately frozen at −80°C until use. For *in vitro* assays with progesterone, a dose-response curve was performed first (data not shown). The uteri were incubated with different concentrations of progesterone [67] and the modulation of nitric oxide levels induced by LPS was determined in the culture supernatants. From this experiment, it was decided to use the lowest effective concentration of progesterone: 50 ng/ml.

### Nitrate and Nitrite Assay

To assess levels of nitric oxide, 24 h cultures with explants of uterus were performed. The accumulated nitric oxide was determined in culture supernatants as nitrate (NO_3_^−^) and nitrite (NO_2_^−^) by Griess technique [21]. The results are expressed as µM NO_2_^−^/mg tissue.

### PCR Analysis

Total RNA from uterine explants was isolated using Trizol reagent according to the manufacturer’s recommendations (Invitrogen, California, USA). Following extraction, RNA was quantified and cDNA was synthesized from total RNA (3 µg) using M-MLVRT, random primers and ribonuclease inhibitor. PCR was performed with specific primers designed using the Primer3 Software [68]. The primers used for LIF were 5′-GGAGTCCAGCCCATAATGAA-3′ (sense) and 5′-TGAGCTGTGCCAGTTGATTC-3′ (antisense) and yielded a product of 184 bp. PCR cycle parameters were as follows: 94°C for 5 min, 35 cycles of : 94°C for 40 s, 57°C for 30 s and 72°C for 1 min, 72°C for 5 min. β-actin was also amplified. Products were loaded onto 2% agarose gel stained with ethidium bromide and bands were visualized on a transilluminator under UV light. Photographs were taken with a digital camera (Olympus C-5060) and analyzed with the Image J software package.

### Radioimmunoassay

Progesterone was measured in serum extracted from LPS-treated mice and control mice sacrificed 6, 12 and 24 h after treatment. Blood was allowed to clot and was centrifuged at 655 g for 10 min. Progesterone was measured by radioimmunoassay as in [69]. Values are expressed as ng progesterone/ml serum.

### Drugs and Chemicals

[1,2,6,7-^3^H(N)]-Progesterone (specific activity: 101.3 Ci/mmol) was provided by PerkinElmer Life and Analytical Sciences Inc. (Walthman, MA, USA). LPS from *Escherichia coli* 05:B55 and progesterone were purchased from Sigma Chemical Co. (St Louis, MI, USA). PCR reagents were provided by Invitrogen Life Technologies Co. (Carlsbad, CA, USA). DMEM, fetal calf serum and antibiotics were purchased from GIBCO (Rockville, MD, USA). Progesterone antiserum for RIA was provided by G. D. Niswender (Colorado State University, Fort Collins, CO, USA). Murine recombinant LIF protein (LIF2010) and LIF polyclonal antibody (#AB1888, for immunohistochemical and western blot analysis) were purchased from Millipore Co. (Billerica, MA, USA). RU-486 was provided by BIOMOL International (Plymouth Meeting, PA, USA). The western blot reagents were obtained from BIO-RAD Laboratories (CA, USA). Secondary antibody was from Jackson ImmunoResearch Laboratories, Inc. (West Grove, PA, USA). The PR polyclonal antibody (C-19, #sc-538) for western blot was from Santa Cruz Biotechnology, Inc. (Santa Cruz, CA, USA). The PR monoclonal antibody (Clon Y85, #323R-16) for immunohistochemistry was from Cell Marque Corporation (Rocklin, CA, USA). All the other chemicals were of analytical grade.

### Statistical Analysis

Treatments were assigned completely random to experimental units. Data were analyzed by means of ANOVA procedures and means were compared by Tukeys test. Differences between means were considered significant when p was 0.05 or less. Different letters indicate significant differences between means. Normality and Homogeneity of variances were tested by Shapiro-Wilks (modified) and Levene test, respectively. In each figure, dissimilar letters denote significantly different values. Statistical analysis was performed using the Infostat Software (Grupo Infostat, FCA, Universidad Nacional de Córdoba, Argentina).
